# Burden of First Osteoporotic Hip Fracture in Spain: A Prospective, 12-Month, Observational Study

**DOI:** 10.1007/s00223-016-0193-8

**Published:** 2016-10-14

**Authors:** Jose Ramón Caeiro, Agustí Bartra, Manuel Mesa-Ramos, Íñigo Etxebarría, Jorge Montejo, Pedro Carpintero, Francesc Sorio, Sonia Gatell, Andrea Farré, Laura Canals, on behalf of the PROA investigators, Antonio Fernández-Cebrián, Eduardo Vaquero-Cervino, José Ramón Caeiro, Emilio Calvo, Íñigo Etxebarría-Foronda, Manuel Bravo-Bardají, Pere Mir-Batlle, Luís-Ángel Montero-Furelos, Gaspar de la Herran-Núñez, Francisco José Tarazona-Santabalbina, Rubén Goñi-Robledo, Naiara Gorostiaga-Perez, Miguel-Angel Valero-Queralt, Guillermo Sánchez-Inchausti, Isabel Serralta-Davia, Joaquín Rodríguez-Miralles, Xavier Granero-Xiberta, Fátima Brañas-Baztán, Vicente Climent-Peris, Núria Galofré-Álvaro, Ana Serrado-Iglesias, Josefa Torres-Martínez, Agustí Bartra-Ylla, Susana Alonso-Güemes, Pedro Carpintero-Benítez, Alberto Delgado-Martínez, Leocadio Rodríguez-Mañas, Cristina Alonso-Bouzón, Olga Laosa-Zafra, Jorge Montejo-Sancho, Vicente Molero-García, Manuel Mesa-Ramos, Luís-Javier Roca-Ruíz

**Affiliations:** 1 Servicio de COT, Complejo Hospitalario Universitario de Santiago, Calle Choupana s/n, ES-15702 Santiago De Compostela, A Coruña Spain; 2 Hospital Universitari Mútua de Terrassa, Plaça del Dr. Robert 5, ES-08221 Terrassa, Barcelona Spain; 3 Hospital Valle de los Pedroches, Calle de Juan del Rey Calero s/n, ES-14400 Pozoblanco, Córdoba Spain; 4 Hospital Alto Deba, Calle Nafarroa Etorbidea 16, ES-20500 Arrasate-Mondragón, Guipúzcoa Spain; 5 Hospital Universitario Fundación Alcorcón, Calle Budapest 1, ES-28922 Alcorcón, Madrid Spain; 6 Hospital Reina Sofía de Córdoba, Avda. Menéndez Pidal s/n, ES-14004 Córdoba, Spain; 7 Amgen S.A., World Trade Center Barcelona, Moll de Barcelona s/n, Edif. Sud, Planta 7, ES-08039 Barcelona, Spain

**Keywords:** Osteoporosis, Hip fracture, Quality of life, Autonomy, Cost

## Abstract

The purpose of this study was to estimate the burden of osteoporotic fractures beyond the hospitalization period covering up to the first year after the fracture. This was a prospective, 12-month, observational study including patients aged ≥65 years hospitalized due to a first low-trauma hip fracture, in six Spanish regions. Health resource utilization (HRU), quality of life (QoL) and autonomy were collected and total costs calculated. Four hundred and eighty seven patients (mean ± SD age 83 ± 7 years, 77 % women) were included. Twenty-two percent of patients reported a prior non-hip low-trauma fracture, 16 % were receiving osteoporotic treatment at baseline, and 3 % had densitometry performed (1.8 % *T*-score ≤−2.5). Sixteen percent of patients died (women 14 %; men 25 %; *p* = 0.0011) during the first year. Mean hospital stay was 11.8 ± 7.9 days and 95.1 % of patients underwent surgery. Other relevant HRUs were: outpatient visits in 78 % of patients (mean 9.2 ± 9.7); walking aids, 58.7 %; rehabilitation facilities, 35.5 % (28.7 ± 41.2 sessions); and formal and informal home care, 22.2 % (49.6 ± 72.2 days) and 53.4 % (77.1 ± 101.0 h), respectively. Mean direct cost was €9690 (95 % confidence interval: 9184–10,197) in women and €9019 (8079–9958) in men. Main cost drivers were: first hospitalization episode (women €7067 [73 %]; men €7196 [80 %]); outpatient visits (€1323 [14 %]; €997 [11 %]); and home care (€905 [9 %]; €767 [9 %]). QoL and autonomy showed a marked decrease during hospitalization, not entirely recovered at 12 months (*p* < 0.05 vs. baseline for EQ-5D, Harris hip score and modified Barthel index). In a Spanish setting, osteoporotic hip fractures incur a high societal and economic cost, mainly due to the first hospitalization HRU, but also due to subsequent outpatient visits and home care.

**Electronic supplementary material:**

The online version of this article (doi:10.1007/s00223-016-0193-8) contains supplementary material, which is available to authorized users.

## Introduction

Osteoporosis is characterized by compromised bone strength predisposing to an increased risk of fracture [1]. In the year 2000, there were approximately 9.0 million osteoporotic fractures with the greatest number occurring in Europe (34.8 %) [2]. Osteoporosis and resulting fractures have significant consequences on human health, QoL and societal burden [2]. Hip fractures place a high burden for patients and healthcare systems due to the advanced age of affected patients, the need for complex surgeries and the high impact on patients’ mobility [3]. However, this burden is systematically underestimated since usually only the admission period is considered. Hip fractures are also associated with a high mortality both during hospitalization [3] and following discharge [4].

In Spain, the annual incidence of hip fractures in patients aged ≥65 years has been estimated at 36,000 (90.5 % of all hip fractures) [3], and it is continuously increasing due to an ageing population (increase of 18 % between 1997 and 2008 [5]). There is limited evidence quantifying the burden of hip fractures at the Spanish national and regional levels, taking into account the differences between regional Health Systems, with only three retrospective chart review studies [6–8] and one study extrapolating data from two clinical trials available [9]. Therefore, there is a need for an updated and reliable estimate of the cost of an osteoporotic hip fracture in Spain to help regions in their decision making.

The primary objective of this study was to estimate health resource utilization (HRU) and related costs associated with osteoporotic hip fractures over 12 months in patients of 65 years of age or older in Spain. The secondary objectives were: to describe patients’ characteristics, health-related quality of life (HRQoL), physical functioning and autonomy/dependency from others and the circumstances leading to the hip fracture.

## Patients and Methods

The PROA (PRospective Observational study on burden of hip frActures in Spain) was a prospective, 12-month, observational study. Patients ≥65 years admitted to hospital due to a first osteoporotic hip fracture (defined as fracture due to a low impact or falling from a standing height or less or any mild or moderate trauma not resulting from a fall [10]) were included. The exclusion criteria were: hip fracture secondary to severe trauma (defined as a fall from a height higher than that of a stool, chair or first rung of a ladder, or severe trauma other than a fall), concurrent non-hip fracture, malignancy or primary bone disease, and participation in an interventional trial in the last 6 months. The protocol was approved by an independent ethics committee, and all patients gave written informed consent before enrolment. For patients who suffered from cognitive impairment, informed consent was given by a legal representative and patient-reported data were provided by the representative at each visit.

The study was conducted in six regions (Andalusia, Basque Country, Catalonia, Galicia, Madrid and Valencia) including small (<200 beds), medium (200–500 beds) and large hospitals (>500 beds). Data were collected at baseline (first admission to hospital), hospital discharge and 4 and 12 months post-fracture. At baseline, the following variables were collected: demographic data, fracture risk factors, comorbidities (Charlson comorbidity index [11, 12]) and circumstances of the fall/event leading to the hip fracture. Fracture-associated HRUs were collected at all visits: inpatient care (length of hospital stay, imaging, type of surgery and/or prosthesis, treatment of complications); re-hospitalizations; ambulatory care (number and type of outpatient visits; physician or nurse), home visits (occupational therapist, physician and nurse) and/or telephone support; rehabilitation (number of physiotherapy sessions); walking aids; visits to emergency departments; and formal (social workers, nursing home stay, rehabilitation facility stay) and/or informal home care (relatives or paid worker). HRQoL (EuroQoL-5 dimensions [EQ-5D] questionnaire [13, 14]) and patient autonomy (modified Barthel index [15] and Harris hip score [16]) were also collected at all visits (retrospectively at baseline, in reference to the status prior to the fracture). HRUs at the time of death were not collected.

### Statistical Analysis

The Spanish Healthcare System perspective has been applied, except for the informal home care resources. Unitary costs were obtained from the eSalud database (http://www.oblikue.com/bddcostes) and adjusted to 2012 values. Mean annual costs and 95 % confidence intervals (CI) were calculated (using 1000 bootstrap samples). The cost of informal home care was estimated by applying the official national minimum wage in Spain. Data regarding home support (formal or informal) before the fracture were asked to the patient or proxy responder (e.g. caregiver or relative) at the beginning of the study. The cost associated with hip fracture was computed as the difference between that of care provided before and after the fracture, as utilized in previous studies [17].

Descriptive analyses were provided for each variable at all the study visits. Changes in continuous variables over time were analysed using paired *T* tests. Differences between subgroups of patients were tested using Student’s *T* tests, Mann–Whitney or Chi-squared tests, as applicable. Time to death was summarized using Kaplan–Meier methodology. Survival differences between men/women were evaluated using a univariate Cox regression model. Statistical analyses were performed with the SAS statistical software package (SAS Institute, Inc, Cary, NC).

## Results

### Baseline Characteristics and Circumstances of the Fall Leading to the Hip Fracture

A total of 487 patients (77 % women) were included in 28 Spanish hospitals between 31 March 2011 and 29 June 2012. Of them, 357 (73.3 %) were followed up during 12 months. Most premature discontinuations (77/130, 59.2 %) were due to death.

Table 1 shows the main characteristics of the study cohort. The mean (SD) age of patients with a first osteoporotic hip fracture was similar for both sexes: 83.2 (6.6) and 81.1 (7.0) for women and men, respectively. Around one-third of patients had at least one previous non-hip fracture, of which 59.7 % had been reported as low impact fractures. A total of 15.6 % of patients were receiving osteoporotic treatment at the time of the fracture occurrence, and only 3 % had undergone bone densitometry testing (1.8 % had BMD *T*-score ≤−2.5).

**Table 1:** Baseline characteristics and osteoporosis risk profile of patients with a first osteoporotic hip fracture in Spain

	Women (*N* = 375)	Men (*N* = 112)	Total (*N* = 487)
Age, years, mean ± SD	83.2 (6.6)	83.1 (7.0)	83.2 (6.7)
≥75 years	339 (90.4)	100 (89.3)	439 (90.1)
Sex, woman	–	–	375 (77.0)
Type of centre			
Small	67 (17.9)	26 (23.2)	93 (19.1)
Medium	101 (26.9)	37 (33.0)	138 (28.3)
Large	207 (55.2)	49 (43.8)	256 (52.6)
Alcohol intake	21 (5.6)	26 (23.2)	47 (9.6)
Active smoking	7 (1.9)	11 (9.8)	18 (3.7)
Body mass index (kg/m^2^)			
<18.5	8 (2.1)	0 (0)	8 (1.6)
18.5–<25.0	159 (42.4)	51 (45.5)	210 (43.1)
25.0–<30.0	112 (29.9)	39 (34.8)	151 (31.0)
≥30.0	58 (15.5)	12 (10.7)	70 (14.4)
Missing	38 (10.1)	10 (8.9)	48 (9.9)
Diagnosis of osteoporosis established by densitometry (*T*-score ≤−2.5)	8 (2.1)	1 (0.9)	9 (1.8)
*T*-score not available	362 (96.5)	110 (98.2)	472 (96.9)
Secondary osteoporosis^a^	10 (2.7)	4 (3.6)	14 (2.9)
Prior non-hip fracture	144 (38.4)	37 (33.3)	181 (37.2)
Prior non-hip fracture by low impact trauma	88 (23.5)	20 (17.9)	108 (22.2)
Time since last fracture, months, median (Q1, Q3)^b^	42.1 (18.7, 109.5)	75.8 (28.2, 163.7)	43.0 (20.4, 123.4)
Location of previous fractures^c^			
Wrist	50 (13.3)	10 (8.9)	60 (12.3)
Shoulder	24 (6.4)	4 (3.6)	28 (5.7)
Spine	16 (4.3)	7 (6.3)	23 (4.7)
Upper arm	17 (4.5)	2 (1.8)	19 (3.9)
Other	67 (17.9)	16 (14.3)	87 (17.9)
Prior osteoporotic treatment	70 (18.7)	6 (5.4)	76 (15.6)
Other risk factors for fracture			
Parental hip fracture	21 (5.6)	9 (8.0)	30 (6.2)
Use of glucocorticoids	22 (5.9)	5 (4.5)	27 (5.5)
Diagnosis of rheumatoid arthritis	13 (3.5)	1 (0.9)	14 (2.9)
Main comorbidities			
Diabetes	75 (20.0)	24 (21.4)	99 (20.3)
Dementia	44 (20.5)	18 (16.1)	95 (19.5)
Cerebrovascular disease	54 (14.4)	28 (25.0)	82 (16.8)
Congestive heart failure	45 (12.0)	13 (11.6)	58 (11.9)
Peripheral vascular disease	48 (12.8)	8 (7.1)	56 (11.5)
Chronic pulmonary disease	22 (5.9)	26 (23.2)	48 (9.9)
Myocardial infarction	29 (7.7)	19 (17.0)	48 (9.9)
Any tumour	13 (3.5)	8 (7.1)	21 (4.3)
Moderate or severe renal disease	13 (3.5)	8 (7.1)	21 (4.3)
Charlson index, mean (SD)^d^	1.8 (1.1)	2.4 (1.7)	1.9 (1.3)
Hip fracture result of a fall	373 (99.5)	110 (98.2)	483 (99.2)
Living arrangements prior to the fall			
Alone in own home	82 (21.9)	11 (9.8)	93 (19.1)
Partner/family member sharing own home	219 (58.4)	79 (70.5)	298 (61.2)
Nursing home	40 (10.7)	15 (13.4)	55 (11.3)
Relatives home	33 (8.8)	7 (6.3)	40 (8.2)
Patient alone at the time of a fall	172 (45.9)	38 (33.9)	210 (43.1)
Where fall happened			
Inside	294 (78.4)	84 (75.0)	378 (77.6)
Outside	79 (21.1)	26 (23.2)	105 (21.6)
Missing	2 (0.5)	2 (1.8)	4 (0.8)
If fall happened outside, weather conditions			
Dry	68 (18.1)	21 (18.8)	89 (18.3)
Wet	10 (2.7)	4 (3.6)	14 (2.9)
Icy	1 (0.3)	1 (0.9)	2 (0.4)
Season when the fall took place			
Winter	59 (15.7)	19 (17.0)	78 (16.0)
Spring	74 (19.7)	28 (25.0)	102 (20.9)
Summer	103 (27.5)	28 (25.0)	131 (26.9)
Autumn	137 (36.6)	35 (31.3)	172 (35.3)
Missing	2 (0.5)	2 (1.7)	4 (0.8)
Patient receiving medications that increase the risk of falls	136 (36.3)	35 (31.3)	171 (35.1)

Data are number of patients (percentage) except when otherwise indicated; ^a^ defined as conditions such as type I diabetes, osteogenesis imperfecta, untreated long-standing hyperparathyroidism, hypogonadism or premature menopause, chronic malnutrition, or malabsorption and chronic liver disease; ^b^ calculated at enrolment in patients with a previous non-hip fracture; ^c^ subjects could have multiple previous fractures at different locations; subjects with more than one fracture in the same location were counted only once in that location; ^d^ valid *N* = 256/86/342 for women, men and overall, respectively; *Q*1 = 25th percentile; *Q*3 = 75th percentile; *SD* standard deviation

The majority of patients lived with a partner or family member sharing their own home (61.2 %), with 19.1 % living alone, 11.3 % living in a nursing home and 8.2 % living in a relative’s home. The circumstances of the fall leading to the hip fracture were similar between men and women. Most falls occurred inside, in the morning and in autumn or summer. Approximately one-third (35.1 %) of subjects were receiving medications that increase the risk of falls (Table 1).

During the follow-up, 18 (3.7 %) patients had at least one new fracture (total of 19 fractures, 95 % osteoporotic origin).

### Health-Related Quality of Life and Patient Autonomy

The HRQoL results and changes in patient autonomy showed a statistically significant decrease during hospitalization and up to 12 months after (Table 2). Furthermore, patients living independent of caregivers or family members decreased after 12 months compared to baseline (36 vs. 77, respectively) (Online Resource 1).

**Table 2:** Changes in health-related quality of life and patient autonomy during the 12-month follow-up

	Baseline (prior to the fracture)	Discharge	4 months	12 months
EQ-5D, health state index, mean (SD)^a^	0.57 (0.39)	0.04 (0.39)*	0.47 (0.41)*	0.53 (0.41)*
Valid *N*	454	446	303	318
Change from baseline, mean (95 % CI)		−0.54 (−0.58 to −0.50)	−0.11 (−0.16 to −0.06)	−0.06 (−0.11 to −0.01)
Harris hip score, mean (SD)^b^	74.9 (19.6)	46.6 (14.6)*	64.7 (17.9)*	69.1 (18.9)*
Valid *N*	353	341	223	244
Change from baseline, mean (95 % CI)		−28.3 (−30.4 to −28.3)	−9.9 (−12.6 to −7.2)	−7.1 (−9.7 to −4.5)
Modified Barthel index, mean (SD)^c^	77.5 (26.9)	40.4 (24.3)*	66.4 (31.4)*	70.4 (31.1)*
Valid *N*	441	433	287	306
Change from baseline, mean (95 % CI)		−37.3 (−39.5 to −35.1)	−12.2 (−14.9 to −9.5)	−9.8 (−12.5 to −7.1)

^a^The health state index score ranges between −0.594 and 1.0. A higher score indicates a more preferred health status, ^b^ Harris hip score ranges between 0 and 100. A higher score indicates better function, ^c^ the modified Barthel index ranges between 0 and 100. A higher score indicates better function

* *p* < 0.05 versus baseline

### Health Resource Utilization

HRU was high, both during the first hospitalization and at 12-month follow-up. The results were similar across genders, except for re-hospitalizations which were more frequent among women versus men (6.4 vs. 3.6 %).

The 95.1 % of patients underwent surgery, mainly intramedullary nail osteosynthesis in women and partial prosthesis in men. Mean length of hospital stay during first hospitalization was 11.8 ± 7.9 days (Tables 3, 4).

**Table 3:** Health resource utilization

	Women (*N* = 375)	Men (*N* = 112)	Total (*N* = 487)
*First hospitalization*			
Hospital stay, days, mean (SD)	11.8 (7.9)	11.9 (8.1)	11.8 (7.9)
Median (min–max)	10.0 (1–69)	10.0 (2–54)	10.0 (1–69)
Geriatric ward, %	0.5	1.7	0.8
Days, mean (SD)	5.0 (1.4)	12.0 (2.8)	8.5 (4.4)
Intensive care, %	26.7	22.3	25.7
Days, mean (SD)	1.0 (0.2)	1.5 (2.0)	1.1 (0.9)
Orthopaedic ward, %	99.2	98.2	99.0
Days, mean (SD)	11.4 (7.7)	11.4 (8.0)	11.4 (7.8)
Other wards, %	2.1	0.8	3.7
Days, mean (SD)	2.9 (5.3)	1.7 (1.6)	2.2 (3.6)
Surgical intervention, %	95.7	92.9	95.1
Intramedullary nail osteosynthesis	45.3	31.3	42.1
Sliding screw osteosynthesis	17.6	17.9	17.7
Partial prosthesis	28.5	36.6	30.4
Total prosthesis	4.8	8.0	5.5
Imaging	96.8	99.1	97.3
CT, %	5.9	6.2	6.0
Num. times used, mean (SD)	1.2 (0.5)	1.0 (0)	1.2 (0.5)
Ultrasound, %	4.3	4.5	4.3
Num. times used, mean (SD)	1.1 (0.3)	1.4 (0.9)	1.1 (0.5)
X-ray, %	96.8	99.1	97.3
Num. times used, mean (SD)	4.0 (1.8)	4.2 (1.7)	4.0 (1.8)
Other procedures^a^, %	0.5	58.9	49.9
Num. times used, mean (SD)	5.8 (3.5)	6.4 (5.0)	5.9 (3.9)
Emergency room visit prior to hospitalization, %	86.9	84.8	86.4
*12-month follow-up*			
Re-hospitalizations, %	6.4	3.6	5.7
Hospital stay, days, mean (SD)	16.2 (13.9)	5.8 (4.5)	14.7 (13.4)
Median (min–max)	12.5 (2–56)	4.5 (2–12)	10.5 (2–56)
Imaging, %	5.9	3.6	5.3
Surgical intervention, %	0.8	0	0.6
Intramedullary nail osteosynthesis	0.5	0	0.4
Partial prosthesis	0.3	0	0.2
Other procedures^a^, %	4.3	3.6	4.1
Ambulatory care			
Outpatient visits, %	81.3	67.0	78.0
Number of visits, mean (SD)	9.1 (9.5)	9.4 (10.9)	9.2 (9.7)
Median (min–max)	6.0 (1–75)	6 (1–58)	6 (1–75)
Nurse at health centre visits, %	31.7	31.2	31.6
Number of visits, mean (SD)	3.2 (4.9)	1.9 (1.4)	2.9 (4.4)
Nurse’s home visits, %	39.5	33.0	38.0
Number of visits, mean (SD)	5.8 (7.1)	7.1 (8.5)	6.0 (7.4)
Physician at health centre visits, %	38.7	38.4	38.6
Number of visits, mean (SD)	3.0 (2.4)	2.8 (2.3)	3.0 (2.4)
Specialist, %	61.9	57.1	60.8
Number of visits, mean (SD)	3.1 (2.5)	2.8 (3.0)	3.0 (2.6)
Physician’s home visits, %	29.1	15.2	25.9
Number of visits, mean (SD)	3.4 (3.8)	4.6 (4.3)	3.6 (3.9)
Rehabilitation facility, %	36.3	33.0	35.5
Number of sessions, mean (SD)	28.5 (43.2)	29.6 (33.5)	28.7 (41.2)
Median (min–max)	16 (1–320)	14 (1–128)	15 (1–320)
Health centre, %	14.7	11.6	14.0
Number of sessions, mean (SD)	27.4 (25.3)	36.8 (38.5)	29.2 (28.2)
Home, %	24.5	25.9	24.8
Number of sessions, mean (SD)	25.7 (41.1)	21.3 (23.9)	24.7 (37.6)
Imaging, %	4.0	1.8	3.5
Num. times used, mean (SD)	6.5 (4.7)	6.0 (5.7)	6.4 (4.6)
Other procedures^a^, %	2.4	1.8	2.3
Num. times used, mean (SD)	2.0 (1.7)	2.5 (2.1)	2.1 (1.6)
Ambulance use, %	53.3	37.5	49.7
Num. times used, mean (SD)	5.0 (10.6)	4.0 (4.4)	4.8 (9.8)
Visits to emergency room, %	16.0	14.3	15.6
Num. times used, mean (SD)	1.9 (2.2)	1.4 (0.7)	1.8 (2.0)
Walking aids, %	60.3	53.6	58.7
Walker	49.9	44.6	48.7
Wheelchair	16.5	14.3	16.0
Home care			
Formal, %	23.7	17.0	22.2
Days, mean (SD)	47.1 (66.7)	61.6 (95.1)	49.6 (72.2)
Median (min–max)	26.5 (1–411)	47.0 (1–618)	25.1 (1–411)
Care from social workers, %	4.5	5.4	4.7
Days, mean (SD)	6.6 (8.2)	5.8 (2.8)	6.4 (7.2)
Nursing home, %	8.8	4.5	7.8
Days, mean (SD)	56.7 (63.5)	110.0 (96.9)	63.7 (69.6)
Rehabilitation facility, %	15.5	10.7	14.4
Days, mean (SD)	38.1 (38.0)	48.7 (45.3)	39.9 (39.2)
Informal, %	56.5	42.9	53.4
Hours, mean (SD)	78.0 (103.7)	73.1 (89.3)	77.1 (101.0)
Median (min–max)	35 (1–672)	38 (2–336)	35 (1–672)
Cared by relatives, %	49.3	39.3	47.0
Hours, mean (SD)	64.7 (81.7)	61.8 (79.4)	64.1 (81.1)
Paid worker, %	24.5	16.1	22.6
Hours, mean (SD)	49.6 (69.9)	44.0 (67.3)	48.7 (69.2)

Mean (SD) number of each HRU calculated among those patients reporting 1 or more

^a^Mainly blood tests; *CT* computed tomography, *SD* standard deviation

**Table 4:** Direct medical costs during the first year after a first osteoporotic hip fracture

Mean € (95 % CI)	Women (*N* = 375)	Men (*N* = 112)
Total direct cost	9690 (9184, 10,197)	9019 (8079, 9958)
*First hospitalization*		
First hospitalization	7067 (6733, 7401)	7196 (6522, 7870)
Hospital stay	4796 (4469, 5122)	4856 (4240, 5472)
Geriatric ward	11 (0, 26)	88 (0, 211)
Intensive care	154 (127, 181)	184 (66, 301)
Orthopaedic ward	4631 (4311, 4950)	4584 (3964, 5205)
Surgical intervention	2064 (1997, 2131)	2128 (1969, 2288)
Intramedullary nail osteosynthesis	795 (706, 884)	545 (393, 697)
Sliding screw osteosynthesis	401 (312, 490)	401 (239, 562)
Partial prosthesis	691 (580, 803)	887 (667, 1106)
Total prosthesis	177 (97, 257)	296 (108, 484)
Imaging	89 (83, 94)	96 (87, 106)
Computed tomography	6 (3, 8)	5 (1, 9)
Ultrasound	4 (2, 5)	5 (0, 10)
X-ray	80 (76, 84)	87 (80, 94)
Emergency room visit prior to hosp.	118 (114, 123)	115 (106, 125)
*12-month follow-up*		
Re-hospitalization^b^	395 (173, 617)	59 (0, 120)
Ambulatory care	1323 (1119, 1528)	997 (753, 1241)
Outpatient visits	329 (291, 367)	281 (204, 359)
Nurse at health centre visits	16 (11, 21)	10 (6, 13)
Nurse’s home visits	73 (56, 91)	75 (40, 110)
Physician at health centre visits	56 (46, 66)	50 (33, 68)
Specialist	122 (106, 138)	104 (72, 135)
Physician’s home visits	62 (46, 78)	43 (16, 70)
Rehabilitation facility	284 (191, 376)	258 (142, 373)
Health centre	48 (32, 65)	52 (12, 91)
Home	235 (148, 323)	206 (100, 313)
Imaging	6 (2, 10)	3 (0, 8)
Ambulance use	486 (336, 635)	269 (157, 382)
Visits to emergency room	42 (26, 57)	29 (14, 44)
Walking aids	177 (148, 207)	157 (104, 210)
Walker	55 (49, 62)	49 (37, 60)
Wheelchair	122 (92, 152)	109 (55, 162)
Home care, mean use	905 (690, 1121)	767 (285, 1250)
Formal	603 (397, 810)	563 (116, 1009)
Care from social workers	18 (5, 31)	18 (3, 35)
Nursing home	258 (129, 387)	254 (−30, 538)
Rehabilitation facility	327 (213, 441)	290 (74, 506)
Informal	302 (236, 368)	205 (114, 296)
Cared by relatives	162 (128, 196)	123 (68, 178)
Paid worker	140 (93, 188)	81 (15, 148)

*CI* confidence interval, *Hosp* hospitalization

There was a large number of outpatient visits (median: 6.0, range: 1–75), use of rehabilitation facilities (median: 15 sessions, range: 1–320), walking aids (58.7 % of patients) and home care (22.2 % of patients with formal care [median of 25 days] and 53.4 % with informal care [median of 35 h]) (Table 3). Seventy-seven patients (15.8 %) required both formal and informal home care.

### Direct Medical Costs

Mean total cost during the first year was €9690 (95 % CI: 9184–10,197) in women and €9019 (8079–9958) in men, with no significant differences between genders except for the cost of re-hospitalizations (Table 4).

The main cost determinant was first hospitalization (€7067 and €7196 in women and men, respectively), followed by ambulatory care and home care (Table 4).

### Subgroup Analyses by Size of Centre

When HRU was analysed by size of centre, large centres showed longer hospital stays (mean of 13.8 days versus 10.2 and 9.0 in small and medium centres, respectively). However, after discharge, patients treated at small centres had more outpatient visits (mean of 10.0 [in all patients] versus 6.3 and 6.5 in medium and large centres), rehabilitation sessions (mean of 17.1 vs. 10.7 and 7.4) and formal home care (mean of 16.6 days vs. 10.5 and 9.3), but less informal care (mean of 29.1 h vs. 48.6 and 41.5 in medium and large centres).

### Mortality

**Fig. 1:**
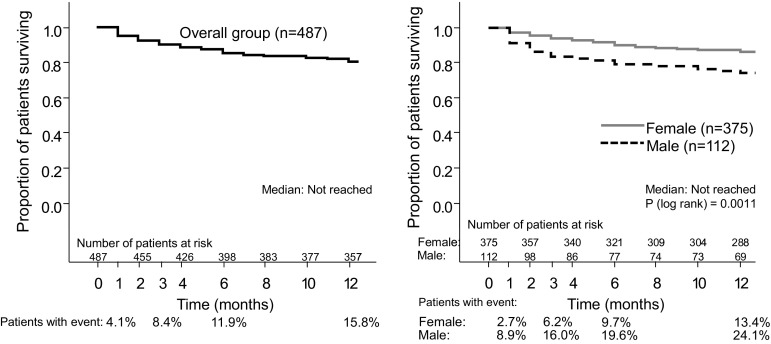
Mortality in the overall group and by gender in the first year after a first osteoporotic hip fracture

During the 12-month follow-up, 15.8 % of patients died, 53 % of them within the first 3 months (Fig. 1). Mortality was significantly higher in men than in women (24.1 vs. 13.4 %, respectively, *p* = 0.0011).

## Discussion

The PROA constitutes the first large, multicentre, prospective study specifically designed to provide estimates on the cost of osteoporotic hip fractures in Spain. Overall, the socio-demographic characteristics of our cohort were comparable to those from similar studies conducted in Belgium [18], Sweden [19] or the UK [20]. Nevertheless, there were some notable differences between this and other national studies. The mean age of this cohort was similar for both sexes and higher than that reported previously, most likely due to the comparatively higher proportion of patients aged 75 years old or above [3, 21, 22]. Furthermore, while the proportion of patients sharing their own home/living alone was similar to that of the Spanish population, the proportion of patients living in a nursing home was greater in this cohort compared to national averages [23]. Lastly, the prevalence of prior vertebral fractures was extremely low (4.7 %) compared to the estimated 20 % in the Spanish population of similar age, most likely due to the fact that in this study only fractures that were documented in the patient’s medical file were collected as opposed to the acquisition of X-rays of the thoracic and lumbar spine using the Genant method [24, 25]. That being said, the similar prevalence of vertebral fractures previously registered in the patient’s file (1.2–4.3 %) reflects the underdiagnosis of these fractures in the daily practice [24].

Of note, almost all first osteoporotic hip fractures occurred in individuals at high risk of fracture, although only a low percentage were previously diagnosed and treated for osteoporosis. The treatment gap (patients eligible for treatment not receiving any drug) for osteoporosis in 2010 was estimated between 57 % (women) and 59 % (men) in the European Union [1]. In Spain, this gap was 25 and 20 %, but in our cohort it could be >30 %, according to the high prevalence of prior osteoporotic fractures and an important underuse of osteoporotic treatments in the recent years [26].

Similarly to previous studies [18, 19, 27], HRU during hospitalization was high, mainly related to a long hospital stay and to the need for surgery. The mean hospital stay (12 days) was similar than that reported in local studies [7] and in a previous analysis of Minimum Basic Data Set between 1997 and 2008 (13 days) [28], but much lower than the 23-day length reported in 1989 [8]. Health resource utilization in the first year following hospital discharge was similar to the observed in Sweden or Belgium [18, 19, 27]. The proportion of patients with re-hospitalization related to the hip fracture was very low in comparison with previous studies that collected all type of hospitalizations (17–30 %) [29, 30].

The cost obtained for the first hospitalization (~€7000) was consistent with the disease-related groups applicable to hip fracture in Spain (210, 211, 236, 558 and 818, cost: €2684-€14,878) [28]. This cost increased by 70 % between 1997 and 2008 in Spain (€4909 to €8365) [8, 28], probably related to the increase in mean age (2 years) and comorbidities of the patients, and the increase in the number of surgical interventions (86 % in 1997) [28].

The total cost in the first year after the first fracture (~€9000) is higher than that reported in Spain after a non-fatal stroke (€4638) but lower than after a myocardial infarction (€19,277) [31]. Our study suggests that, if only the first hospitalization is considered, one-fourth of the total annual cost of a hip fracture might be underestimated.

Compared to other European countries, the cost seems to be approximately a 25 % lower (€13,470 in Belgium; €14,221 in Sweden). In a UK cohort, the cost was slightly lower (€7536) [20], but in that study the costs associated with rehabilitation services and home care were not taken into account.

Mortality was high, especially in males (24.1 %). In both genders, mortality rates were almost three times higher compared to the annual mortality rate of Spanish general population of a similar age (7.4 and 4.5 %, respectively, in males and females of 80–84 years old) [32].

Prior to the fracture, the HRQoL was similar to that reported in Spanish population aged ≥85 [33], but it showed a marked worsening during the hospital stay and was not entirely resolved after 12 months, highlighting the long-term burden of the hip fracture.

Our study has some limitations. The similarity in age between sexes combined with the high proportion of patients aged 75 years old or above may limit the generalizability of these results to all patients with osteoporotic hip fractures in Spain aged ≥65 years old. The total cost may have been underestimated due to the inability of the study to collect the HRU at the time of death, inherent to the nature of observational design. Patient-reported HRU after hospital discharge, such as visits to the general practitioner, emergency room visits or re-hospitalizations, may have been underestimated due to the inability of the patient to recall information, leading to potential misclassification.

Strengths of our study include the large sample size and the geographically distributed recruitment, which ensures that it represents the regional diversity of Spain. Also, the prospective follow-up allowed a more comprehensive data collection on both the economic and humanistic burden of the condition not routinely included in patients’ medical records.

In conclusion, in a Spanish setting, osteoporotic hip fractures incur a high societal and economic cost, mainly due to the high HRU during the first hospitalization, but also due to subsequent outpatient visits and home care. Hip fractures were also associated with a high mortality of approximately one in six patients during the first year. The high prevalence of known risk factors and the low number of patients receiving prophylactic treatment highlight the undertreatment of this population, typically women older than 75 years with prior fractures, several comorbidities such as diabetes or dementia, and receiving medications that increase the risk of falls. By comparison, men in this study cohort not only received less osteoporosis follow-up prior to the hip fracture, but also exhibited a greater frequency of risk factors such as smoking and excessive alcohol consumption. Together, these results reflect the need for improving the diagnostic and therapeutic management of osteoporosis in Spain.

## Electronic supplementary material

Below is the link to the electronic supplementary material.

Supplementary material 1 (PDF 112 kb)
