# Supplementary material for: Burden of First Osteoporotic Hip Fracture in Spain: A Prospective, 12-Month, Observational Study
Source: Calcif Tissue Int. 2016 Oct 14;100(1):29–39. doi: 10.1007/s00223-016-0193-8 (PMC5214753; doi:10.1007/s00223-016-0193-8)
Supplement: Supplementary file 1 — Supplementary material 1 (PDF 112 kb) [file 223_2016_193_MOESM1_ESM.pdf]

Burden of first osteoporotic hip fracture in Spain: a prospective, observational, 12-month study

Calcified Tissue International

Authors: Jose Ramón Caeiro (1), Agustí Bartra (2), Manuel Mesa-Ramos (3), Íñigo Etxebarría (4), Jorge Montejo (5), Pedro Carpintero (6), Francesc Sorio (7), Sonia Gatell (7), Andrea Farré (7), and Laura Canals (7) on behalf of the PROA investigators

Corresponding author:
Dr. José Ramón Caeiro
Servicio de COT
Complejo Hospitalario de Santiago de Compostela
Calle Choupana s/n, ES-15702 Santiago de Compostela, A Coruña, Spain.
Phone: +34-609458500
Fax: +34-981950900
Email: jrcaeiro@telefonica.net

Supplementary Table 1. Changes over time in living arrangements

	Baseline Living Arrangement					
	Own home alone	Own home with partner / family member	Nursing home	Relatives home	Unknown	Total
	n (%)	n (%)	n (%)	n (%)	n (%)	n (%)
**4 months**						
Own home alone	37 (7.6)	13 (2.7)	2 (0.4)	2 (0.4)	-	54 (11.1)
Own home with partner / family member	29 (6.0)	200 (41.1)	3 (0.6)	12 (2.5)	-	244 (50.1)
Nursing home	12 (2.5)	18 (3.7)	36 (7.4)	4 (0.8)	-	70 (14.4)
Relatives home	4 (0.8)	17 (3.5)	0 (0.0)	14 (2.9)	-	35 (7.2)
Unknown	11 (2.2)	50 (10.3)	14 (2.9)	8 (16.4)	1 (0.2)	84 (17.2)
Total	93 (19.1)	298 (61.2)	55 (11.3)	40 (8.2)	1 (0.2)	487 (100)
**12 months**						

Own home alone	36 (7.4)	15 (3.1)	2 (0.4)	1 (0.2)	-	54 (11.1)
Own home with partner / family member	28 (5.7)	183 (37.6)	5 (1.0)	12 (2.5)	-	228 (46.8)
Nursing home	10 (2.1)	15 (3.1)	26 (5.3)	5 (1.0)	-	56 (11.5)
Relatives home	3 (0.6)	15 (3.1)	0 (0.0)	9 (1.8)	-	27 (5.5)
Unknown	16 (3.3)	70 (14.4)	22 (4.5)	13 (2.7)	1 (0.2)	122 (25.1)
Total	93 (19.1)	298 (61.2)	55 (11.3)	40 (8.2)	1 (0.2)	487 (100)

Percentages based on total number of patients (N=487)
